# Shock impact simulation model along with the harmonic effect of the working device

**DOI:** 10.1038/s41598-024-55122-5

**Published:** 2024-02-23

**Authors:** Andrzej Grządziela, Marcin Kluczyk, Paweł Piskur, Krzysztof Naus

**Affiliations:** Polish Naval Academy, Smidowicza 69, 71-128 Gdynia, Poland

**Keywords:** Mechanical engineering, Applied mathematics

## Abstract

This study analyses the impact of initial conditions on the results of numerical simulations of the fan load with underwater detonation and simultaneously typical harmonic loads from the operating device. It was shown that different initial conditions affect both displacement and velocity results. Furthermore, significant differences were indicated when comparing the results with devices without harmonic loads. The results indicate the need for more detailed analyses at the initial stage of modelling the impact resistance of devices planned for ship installation.

## Introduction

The literature defines survivability as the measure that enables a warship to survive in a militarily hostile environment^1–3^. This work has adopted the following survivability definitions^4^:

- Survivability is a weapon system's capability to continue carrying out its designated mission(s) in a combat environment. Survivability is a function of both susceptibility and vulnerability.
- Susceptibility is the combination of factors determining the probability of being hit by a given threat (such factors include the susceptibility to detection, classification, targeting, attack, being hit).
- Vulnerability is the extent of a system's degradation after being subjected to combat threats, that is, the degree of mission impairment due to sustaining finite levels of damage caused by weapon hit.

During the preliminary design process of ship construction, there is a need for a deeper understanding of ship security analysis^5^. Survivability is the ship's ability to survive obtained using multi-level design methods, including many threats like preliminary design resistance of the UNDEX (underwater explosion) effect^1^. Computer technology is used to exploit initial design approaches which can address innovation early in the ship design process, including the objective of survivability, especially vulnerability. Since a ship's survivability depends on layout, the approach adopted in this research takes advantage of an architecturally orientated simulation model of UNDEX shock resistance^6^. The strategy of integrating vulnerability assessment of the fan foundation is an example of the process of naval architecture. It is the typical task for calculating the resistance of weapon effect.

A ship's survivability to underwater explosions (UNDEX phenomena) is predicted in the initial stages of calculations, usually already on the first or second Evans spiral^7^. Survivability assessment methods currently concern numerical simulation methods and validation tests on impact machines during sea trials on specially prepared ships^8,9^. Since such tests are time-consuming and costly, an analysis of the required detail and an initial analysis of calculation and measurement errors should be carried out. The algorithm prepared in this way reduces the required calculations, the selection of measuring equipment, and the plan of measurement campaigns in laboratories and during sea shock trials. Numerical simulation studies require precise conceptual assumptions, knowledge of materials, dynamic excitation models and an acceptable margin of error^10,11^. This paper indicates the possibility of making errors at the initial stages of the simulation, which may introduce unacceptable errors in the design process. An explanation of the meaning of "the ship's survivability" makes it possible to define three other related concepts: i.e. Susceptibility, Vulnerability and Recoverability. Susceptibility is “the ship's inability to evade the sensors, weapons, and weaponry effects of man-made hostile environment,” which is assessed in three phases: probabilities, i.e. the threat is active, the detecting an enemy, and finally, the probability of a successful hitting the ship^12^. Vulnerability is “the inability of the ship to withstand damage mechanisms from one or more hits, to its vincibility, and to its liability to serious damage or loss when hit by threat weapons”^13^. Recoverability is defined as the “ability of a ship and its crew to restore mission essential functions given a hit by one or more threat weapons”^13^. This work is closely related to the concept of vulnerability, especially in the preliminary design of the foundation of ship machinery and equipment using numerical simulation models.

## Shock wave in UNDEX phenomena in ship’s hull structure

The UNDEX phenomenon is characterised in the literature as a near or far field. For the near field, the explosion is energetically large and occurs close to the ship at such a distance that the maximum radius of the gas bubble can come into contact with the hull's surface^5,14^. In this case, the main effects of the phenomenon are plastic deformation and even cracking of the ship's hull. Secondary phenomena include the possibility of a loss of buoyancy by flooding one or more compartments and a high-energy shock that can cause loss of life to crew members. Far-field explosions occur at such a distance that the gas bubble does not directly impact the ship's hull^15^. The propagation waves in this phenomenon cause elastic or elastoplastic deformation^14^. It should be noted that for large depths of the sea basin, the ship is affected by the first shock wave generated by the explosion and subsequent bubble pulses from the gas pulsation cycle inside the bubbles. The features determining the course of the phenomenon and energy of pulsation are the mass and TNT equivalent of the charge, the depth and the distance of the ship from the epicentre of the detonation. During far-field phenomena, the main concern is the ability to withstand the shock of the ship's internal systems and personnel.

The literature on the design and validation of ships subjected to UNDEX phenomena is quite extensive. It covers many aspects and design phases, for example, NAVSEA 0908-LP-000-3010A, MIL-S-901D, MIL-STD-810, BV043, STANAG 4142, NATO - STANAG 4137, NATO 4549 and others^5,8^. Many researchers have studied the transient response of structures affected by an underwater explosion^10,16^. Studies have focused on submerged body-fluid interactions where plane or spherical waves acted on submerged shells subjected to transient acoustic waves^10,14,15^. Regardless of the obtained results, most authors used and still use the results of experimental research, which are characterised by coefficients characteristic of all equations describing the phenomenon of the maximum and transient pressure of the detonation wave^17–19^.

The basic design concept for ship shock is the shock factor, which is quantified. The literature commonly assumes that W is the weight of the explosive and D is the distance of the blast from the ship; the equation gives the shock coefficient^8,20,21^:

1 $$\begin{array}{*{20}c} {\frac{{W^{n} }}{D}.} \\ \end{array}$$

Various authors present the values of the n factor in a somewhat different way^22–24^; however, the exact values are usually contained in classified documents from shock trials^25,26^.

A typical acceleration caused by UNDEX phenomena *a*(*t*) for a ship has a maximum acceleration *a*_*max*_ that lasts for *T*_*shock*_ ∼$1 \div 2 \times 10^{ - 3 } \;{\text{s}}$. Analysing the problem of shock impulse absorption, let’s consider the vibrational response to the component, excitation *a*(*t*), generated by the detonation pressure impulse during the impact without filtering. In the case of modelling shock absorbers, the maximum acceleration of the element in response to the shock should be predicted in the first phase of simulation tests. Suppose that each mode is forced by a base subjected to a prescribed acceleration *a*(*t*). The basic oscillator equation is:

2 $$\begin{array}{*{20}c} {\ddot{x} + \omega_{0}^{2} x = a\left( t \right),} \\ \end{array}$$

where $\omega_{0} = \sqrt{\frac{k}{m}}$ is the natural frequency of the oscillator of mass *m* and spring constant *k*. Assuming that the (mode) component complies with the Eq. (2), we need to find *x*(*t*) and then calculate the maximum acceleration:

3 $$\begin{array}{*{20}c} {A_{max} = max_{t} \left( {\ddot{a}\left( t \right)} \right)} \\ \end{array}$$

Since we do not know the exact form of *a*(*t*), we can assume that *a*(*t*) is given by an elementary form for:

4 $$\begin{array}{*{20}c} {t \le T_{shock}\, and \;a\left( t \right) = 0} \\ \end{array}$$

otherwise.

The work assumes that a shock wave is a huge, almost discontinuous compressive pressure wave generated by the detonation wave of an explosive charge reaching the outer surface of a gas bubble. Assuming unlimited sea depth, the wave propagates as a spherical wave that initially travels much faster than the speed of sound in the water due to the acceleration from the initial charge explosion. Other theoretical assumptions were adopted similar to the literature^14^. The model omitted the effect of cavitation and overload because it is not as large as the initial impulse, and the time parameters of its occurrence are difficult to model precisely^27^.

The UNDEX similarity equations represent a simplified way of calculating the characteristics of an underwater explosion because the analytical models are very complex, and the obtained results are challenging to validate. The model adopts the “similarity” principle, where the main variables in the equations describing the pressure of the detonation wave are the weight of the charge and the distance of the hull from the epicentre^14^. The authors are aware that the similarity equations used do not directly model the physics of the detonation wave but provide a good understanding of the behaviour of the UNDEX event.

The pressure–time course caused by the shock wave is called the “peak pressure approximation”, and a complete derivation of the formula is provided in the reference^20^. The pressure time waveform due to a shock wave can be written by different formulas. The most popular is as follow^28^:

5 $$\begin{array}{*{20}c} {p\left( t \right) = P_{max} e^{{ - \frac{t}{\theta }}} } \\ \end{array}$$

The maximum pressure can be formulated from the similarity as^28^:

6 $$\begin{array}{*{20}c} {P_{max} = K\left( {\frac{{W^{\frac{1}{3}} }}{R}} \right)^{\alpha } } \\ \end{array}$$

where R is the distance of the measurement point from the epicenter of detonation and W is the weight of the TNT charge. The decay constant θ is defined as the time taken for the pressure to drop to 36.8% of the peak pressure and is defined as^28^:

7 $$\begin{array}{*{20}c} {\theta = W^{\frac{1}{3}} K\left( {\frac{{W^{\frac{1}{3}} }}{R}} \right)^{\alpha } } \\ \end{array}$$

The constants *K* and α are derived experimentally and are specific to a given metric system of units. Differences in the coefficients in formulas (5)–(7) result from tests of explosions caused by explosives of different sizes at different depths, which may cause discrepancies in experimental results. A comparison of the formulas and a commentary on the specifics of research campaigns can be found in the paper^29^.

For numerical simulations of the underwater detonation pressure profile, the model proposed by Constanzo was adopted in this work^30^. The choice of the model is justified by the consistency of the results of the numerical simulation with the results of experimental research carried out by the authors of this work^31^. For further calculations the formula (5) is used for $0 \le t \ll \theta$ and charge explosive TNT type.

8 $$\begin{array}{*{20}c} {P\left( t \right) = P_{max} e^{{ - \frac{t}{\theta }}} } \\ \end{array}$$

The $P_{max}$ is calculated in Imperial system [psi] as follow^5^:

9 $$\begin{array}{*{20}c} {P_{max} = 2.16 \times 10^{4} \left[ {\frac{{\left( {W\left[ {lbs} \right]} \right)^{\frac{1}{3}} }}{{R\left[ {ft} \right]}}} \right]^{1.13} } \\ \end{array}$$

and

10 $$\begin{array}{*{20}c} {\theta \left[ {msec} \right] = 0.06 \cdot \left( {W\left[ {lbs} \right]} \right)^{\frac{1}{3}} \cdot \left[ {\frac{{\left( {W\left[ {lbs} \right]} \right)^{\frac{1}{3}} }}{{R\left[ {ft} \right]}}} \right]^{ - 0.18} } \\ \end{array}$$

## The purpose and initial assumptions for simulation studies

During numerical simulations, it must be determined whether the structural predictions of the ship construction are adequate. To achieve this objective, validation metrics are needed, i.e.:

- Peak acceleration: if the component's characteristic frequency is greater than 6 kHz;
- The product $V_{kick - f} \cdot \omega$, where ω is either the vibration frequency of the analysed device or element,
- For resonance $n \cdot V_{kick - f} \cdot \omega$, where *ω* is the resonant frequency of the component and the excitation frequency of the part of the vessel containing the component, and *n* is the number of periods of the ship oscillating to damp out.

where $V_{kick - f}$—the maximum kick-off velocity after impact^32^.

The pressure described as *p*(*t*) is a function relating the pressure movement of the detonation wave out from epicentre in time and is called "peak pressure approximation". The nature of the function indicates, just like the actual detonation, that the function increases rapidly up to the maximum pressure described as *P*_*max*_ and then decreases exponentially. The course of changes depends on the decay constant θ is defined as the time taken for the pressure to drop to 36.8% of the peak pressure and is defined in formula^10^. *P*_*max*_ is the maximum pressure of the detonation wave (shock front) at a specified distance from the epicentre but outside the radius of the gas bubble. R is the distance of the measurement or simulated point from the epicentre of detonation, and W is the weight of the equivalent TNT charge. The constants *K* and α are derived experimentally and depend on characteristic factors such as the type of charge and detonation depth, but their values are not usually published^30^.

Component testing procedures are designed for fixed reflection, with some parameters uncertain. These uncertainties necessitate validating component test procedures concerning frequencies and damping factors. It should be assessed whether the computer codes can accurately predict the modal frequencies and the damping factors of the modes induced during the ship's shock. Errors make reproducing the accelerations and resonance interactions of the device or component being analysed impossible. The frequencies and attenuations should be predicted accurately enough so critical components do not fail during a shock test due to errors in the component testing simulation procedures. The general problem presented in this paper is the initial conditions of the Ordinary Differential Equations (ODE) w numerical simulations of the UNDEX impacts. The analysis of the many military standardisation regulations for assessing resistance to impacts indicates the application of quite simple models of dynamic loads, no analysis of the effects of successive impacts from energy gas bubbles (the potential resonance) and no assumptions for parallel shock and harmonic loads occurring from working devices in the structure of the ship's hull.

**Figure 1:**
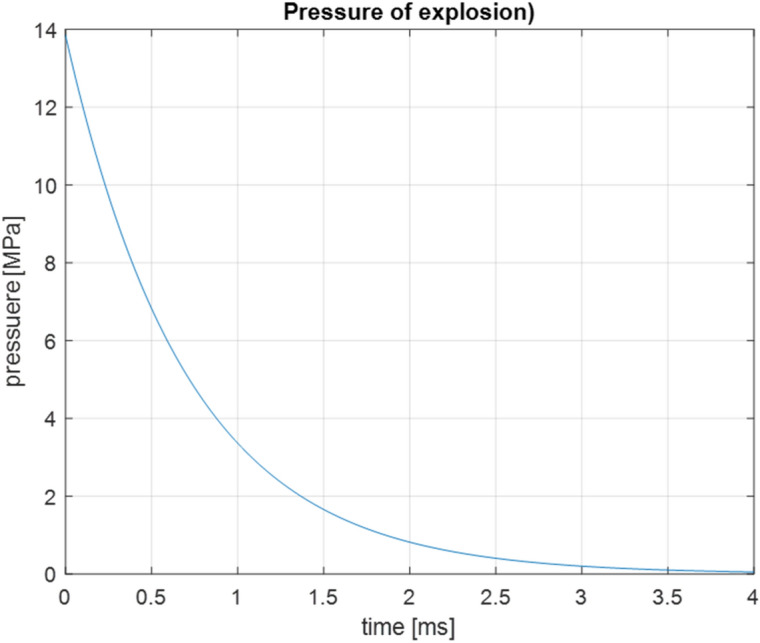
The shock wave time course calculated in simulations.

For the simulation research, it was assumed, in contrast to the simple models proposed in the standardisation regulations, that during the UNDEX phenomenon, the device works and is dynamically loaded with typical harmonic forces on board. The supply fan located on shock absorbers on the bulkhead in the ship's engine control room was adopted as the test object. The mass of the fan is 1.8 kg, the harmonic oscillation of the fan’s shaft equals f = 16 Hz, and the main load harmonic is 48 Hz—3-blade fan. In the simulation, the explosion of a Russian M-26 type fixed moored mine with a TNT load weight of 240 kg was assumed to be the UNDEX impact load. The assumed distance from the epicentre is 20 m, which significantly approximates the results to real conditions. The earlier test studies of ship structure damping made it possible to evaluate the damping on a logarithmic scale. During the tests, vibration accelerations caused by the test shock on the underwater part of the hull were recorded using a logarithmic scale, where signal 1 ($Sign_{1}$) meant the acceleration recorded at the bottom of the ship and signal 2 ($Sign_{2}$) was the acceleration recorded on the fan foundation. Using the formula:

11 $$\begin{array}{*{20}c} {\frac{{\log_{10} Sign_{1} }}{{\log_{10} Sign_{2} }} = \log_{{Sign_{2} }} Sign_{1} } \\ \end{array}$$

the computational response of the foundation to virtual detonation was determined by dependence:

12 $$\begin{array}{*{20}c} {\log_{10} Y_{fan} = \frac{{\log_{10} Y_{shock} }}{{\log_{{Sign_{2} }} Sign_{1} }}} \\ \end{array}$$

where $Y_{shock}$ is the calculated signal from the virtual impact acting on the bottom of the hull, and $Y_{fan}$ is the signal loaded on the fan foundation. For further calculation the shock wave time course was simulated according the formula (8)—Fig. 1. The acceleration is the input value for the SDOF system of the fan.

In the simulation, the explosion of a Russian M-26 type fixed moored mine with a TNT load weight of 240 kg was assumed as the UNDEX impact load^21^. The assumed distance from the epicentre is 20 m, which significantly approximates the results to real conditions. The earlier test studies of ship structure damping made it possible to evaluate the damping on a logarithmic scale. Scaling the results allowed the development of the estimated size of the actual load acting on the test object^31^.

## Simulation results and discussion

**Figure 2:**
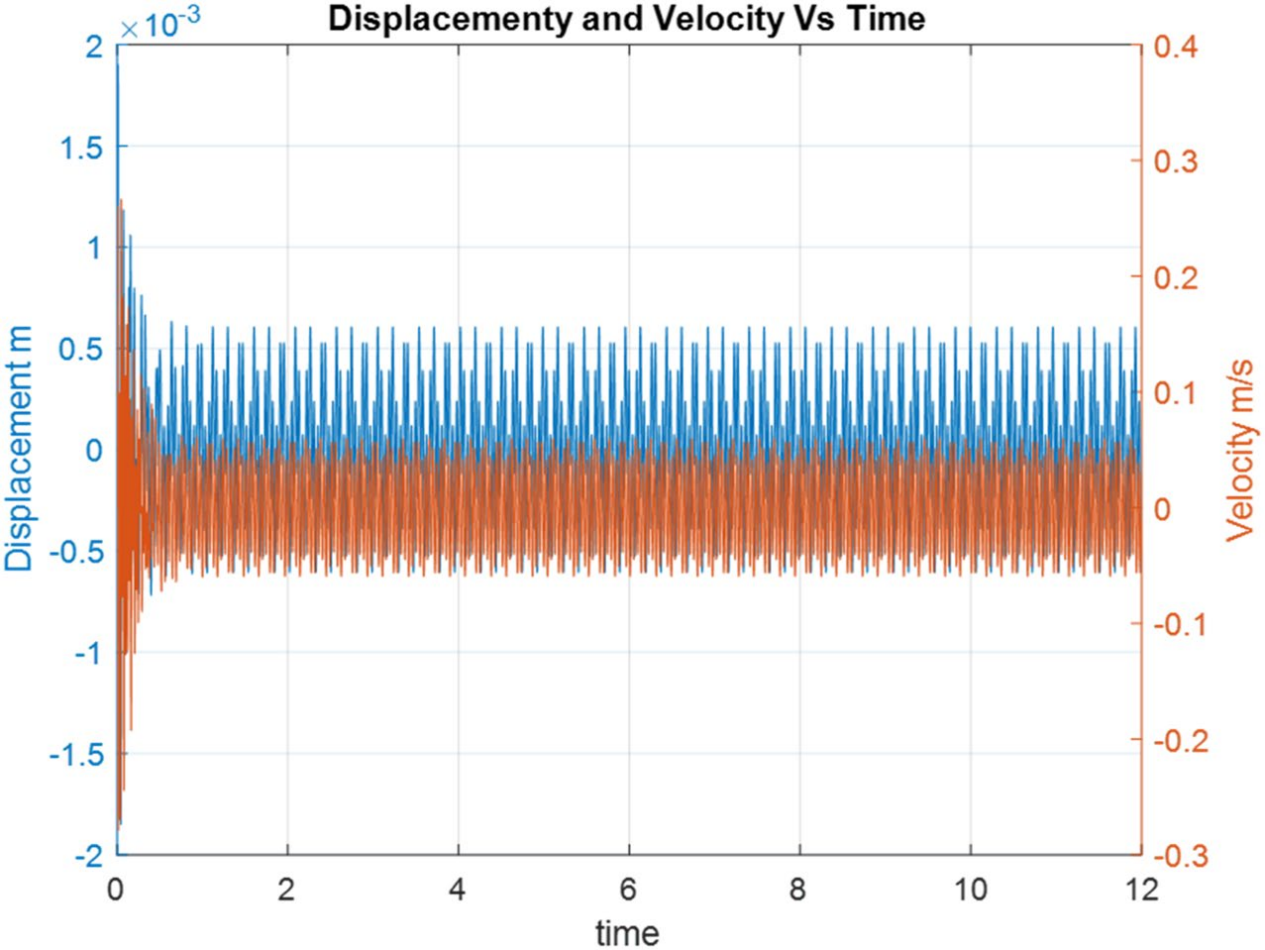
The time history of the working fan’s simulation model.

The first step of the research was preparing a simple ODF dynamic model of the working fan in Matlab code, in which there is a weight load and the residual unbalance is the effect of using three blades. Such a model can be used for SRS tests in later studies. The time history of the working fan’s model is shown in Fig. 2.

The simulation tests' primary purpose was to demonstrate the significant impact of initial conditions on the results of displacements and vibration velocities. A code in Matlab was prepared to enter a precise time for the first and subsequent detonation pulses^14,21^. The sizes of successive pulses in magnitude and periods were introduced based on literature analysis. The following initial conditions were assumed in the tests:

- $\dot{y} = max$ the velocity of the vibration vector is consistent with the detonation pulse vector and has a maximum value,
- $\dot{y} = min$ the velocity of the vibration vector is opposite to the detonation pulse vector and has a maximum but opposite value,
- $y = min$ displacement of vibration has a minimum value; the absorber is compressed,
- $y = max$ displacement of vibration has a maximum value; the absorber is stretched,
- $y = 0$ displacement of vibration has a reference value; the absorber is relaxed.

**Figure 3:**
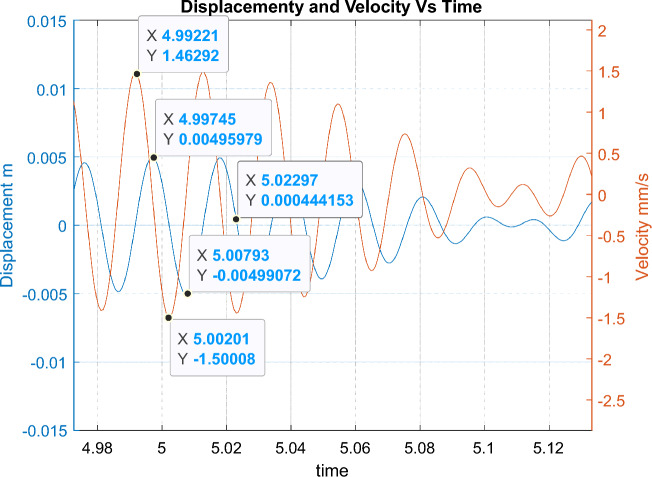
Time points meeting the requirements specified in the initial conditions.

The simulation assumes an explosion time at a point of t = 5 s, and subsequent gas bubble interactions result from the adopted model. By analysing the time waveform of the displacement and the vibration velocity for the operating fan, time points meeting the requirements specified in the initial conditions—Fig. 3 were indicated.

**Figure 4:**
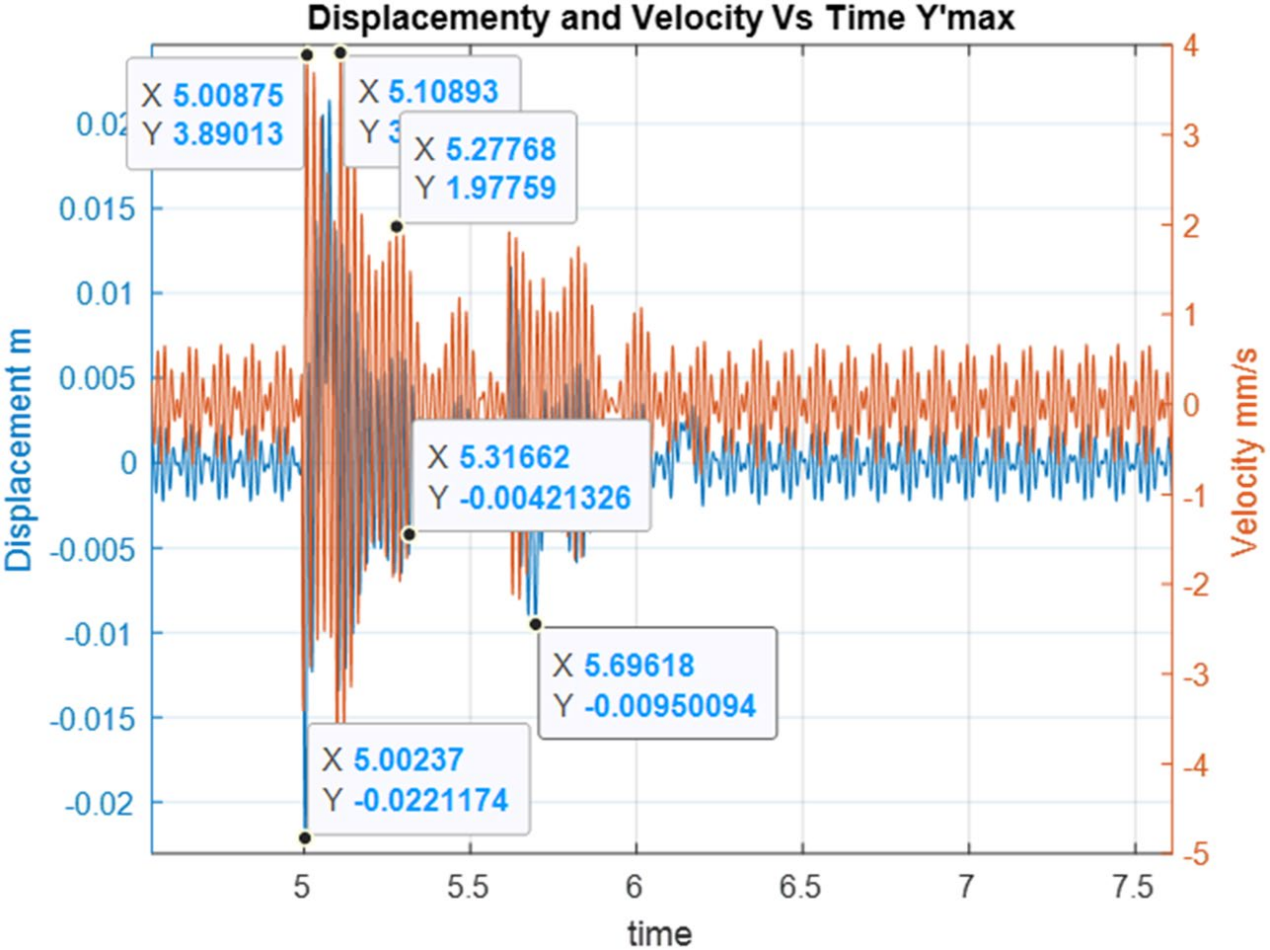
Displacement and velocity for Y’ = max.

**Figure 5:**
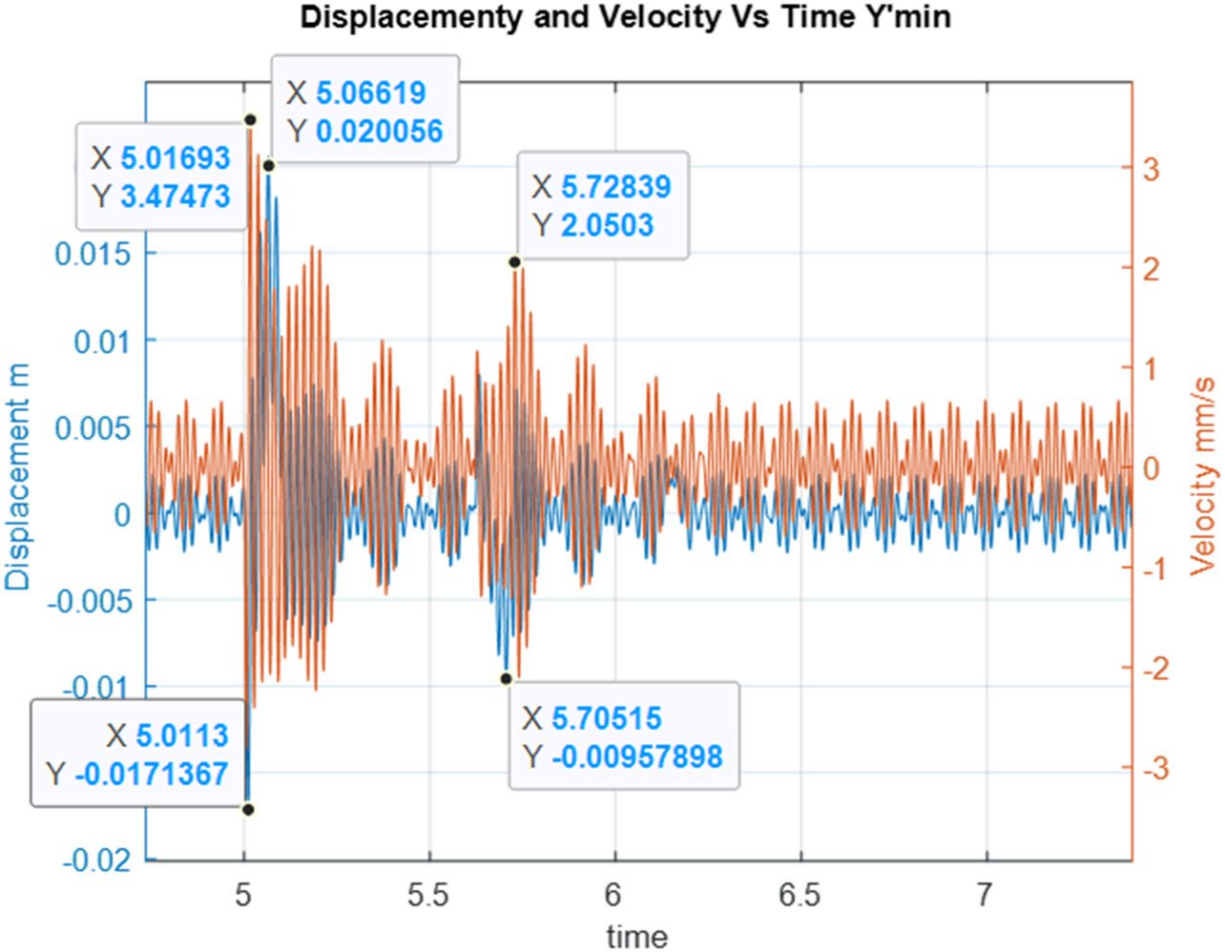
Displacement and velocity for Y’ = min.

**Figure 6:**
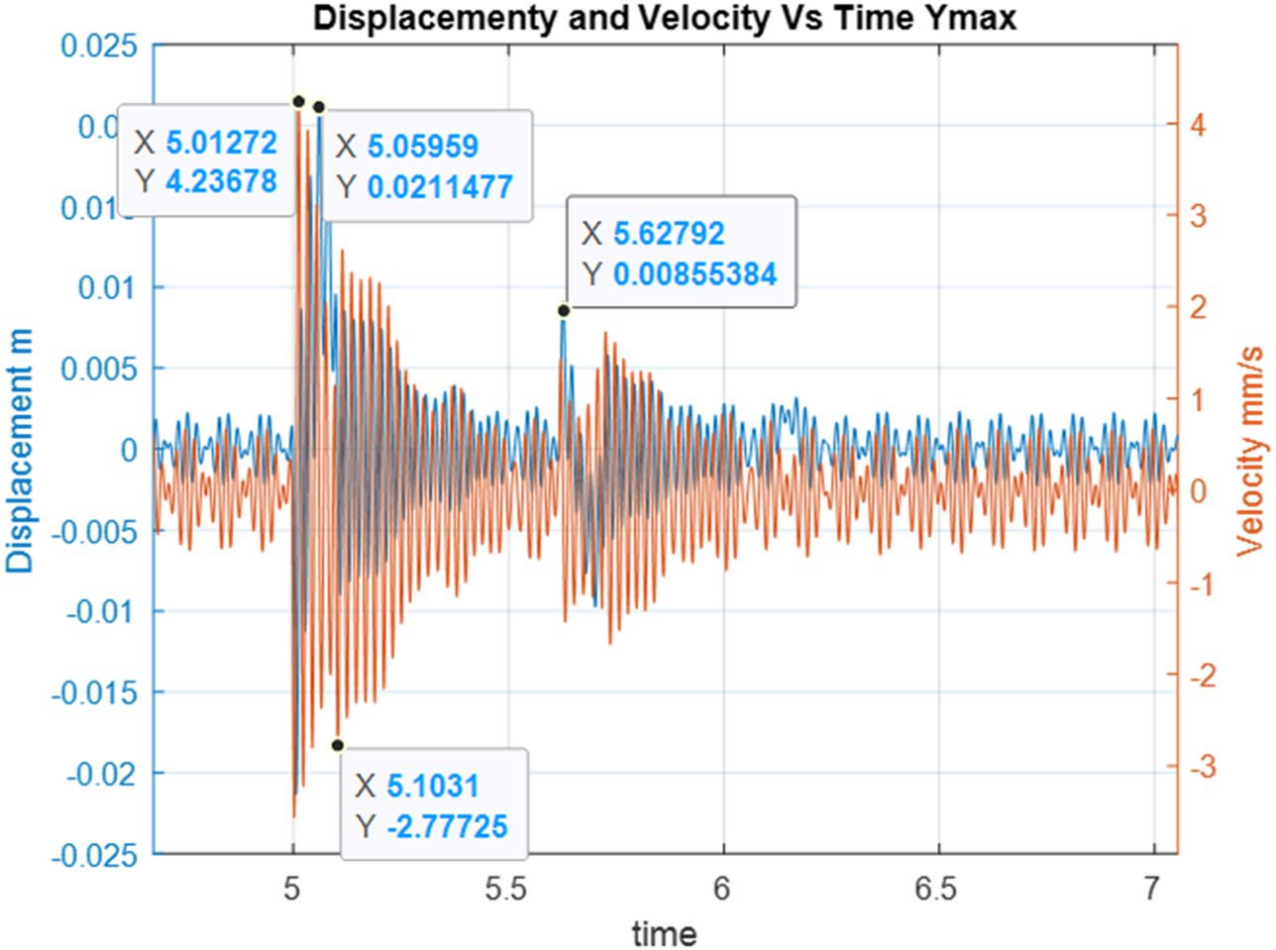
Displacement and velocity for Y = max.

**Figure 7:**
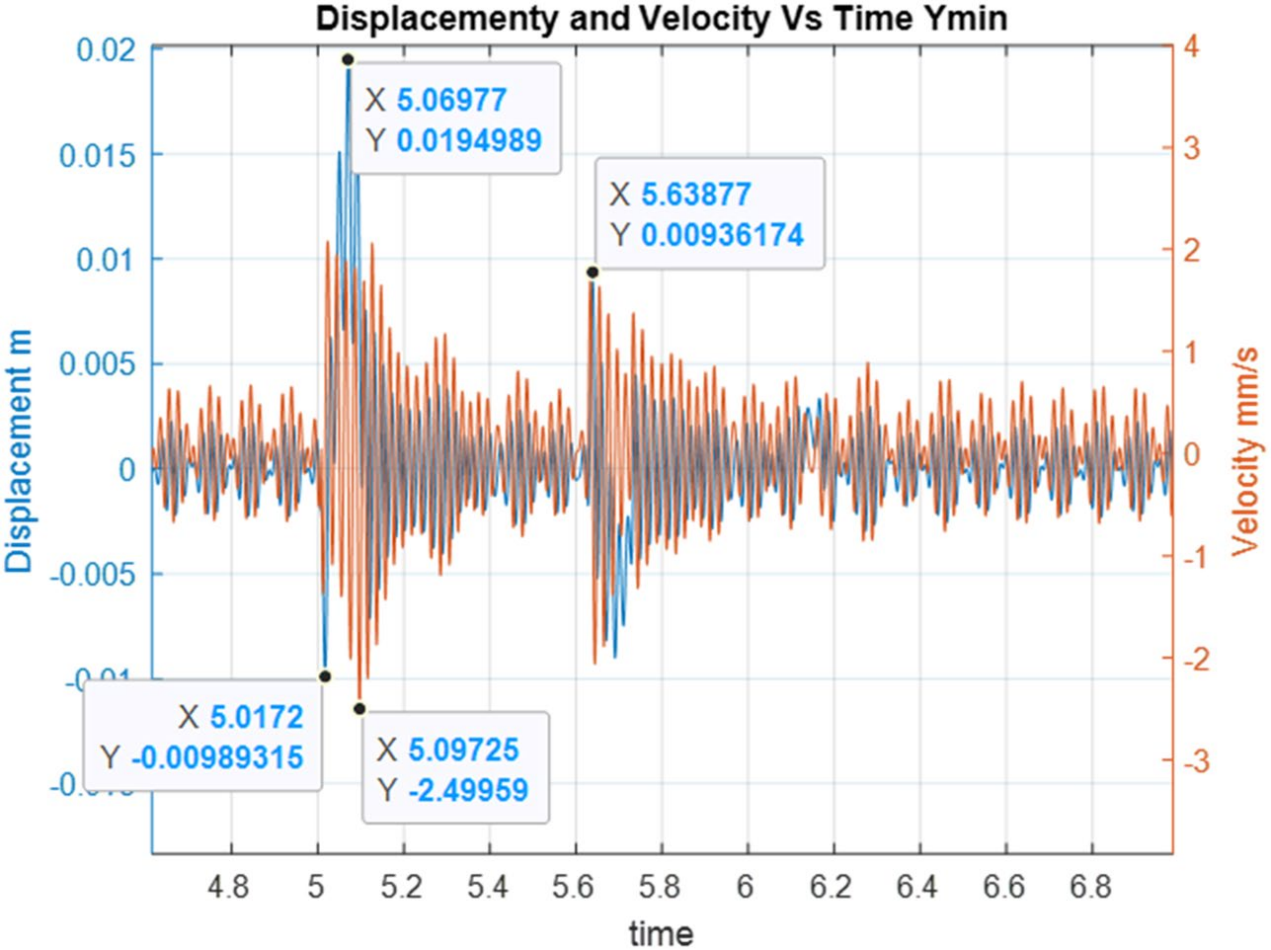
Displacement and velocity for Y = min.

**Figure 8:**
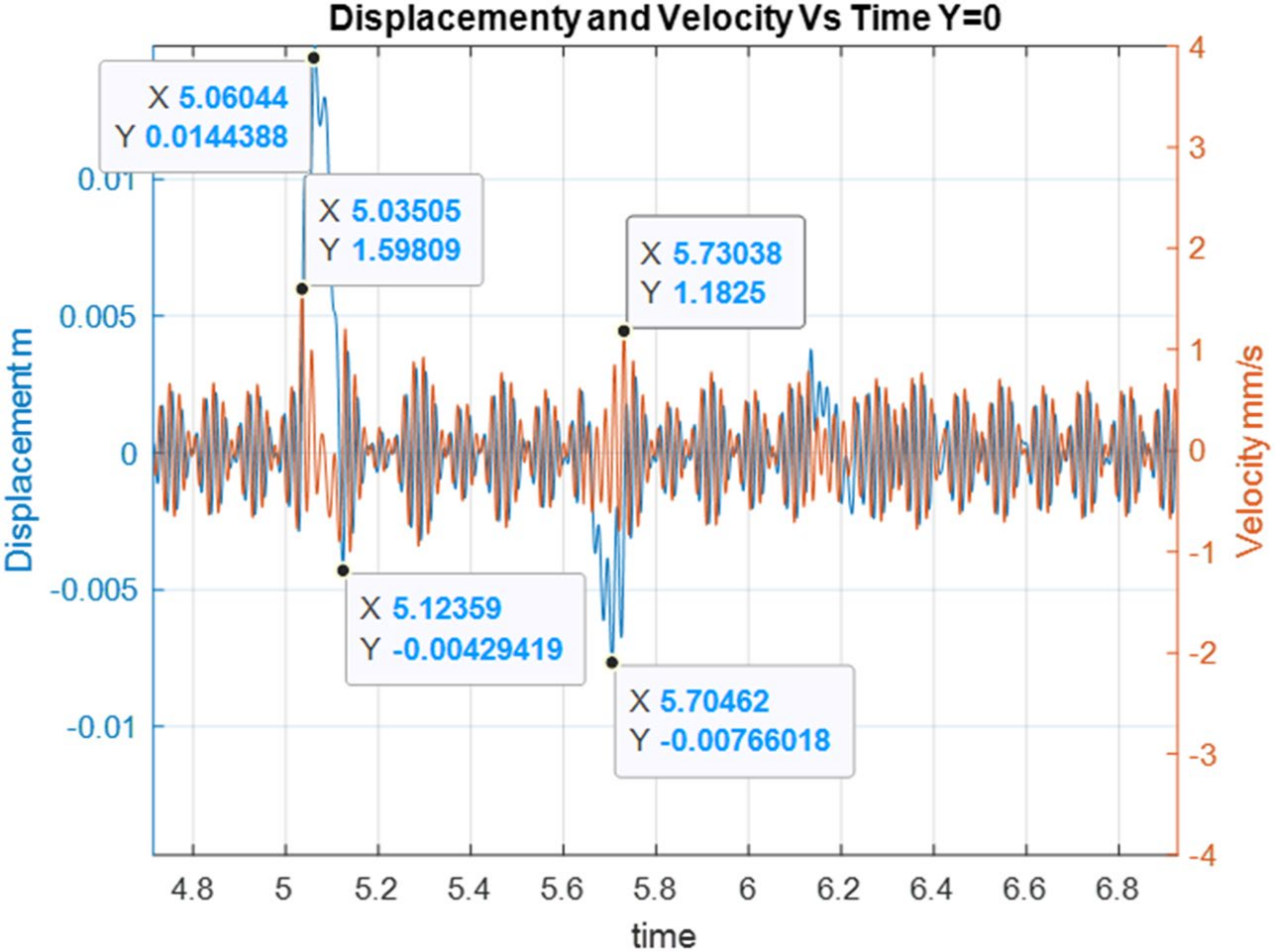
Displacement and velocity for Y = 0.

**Figure 9:**
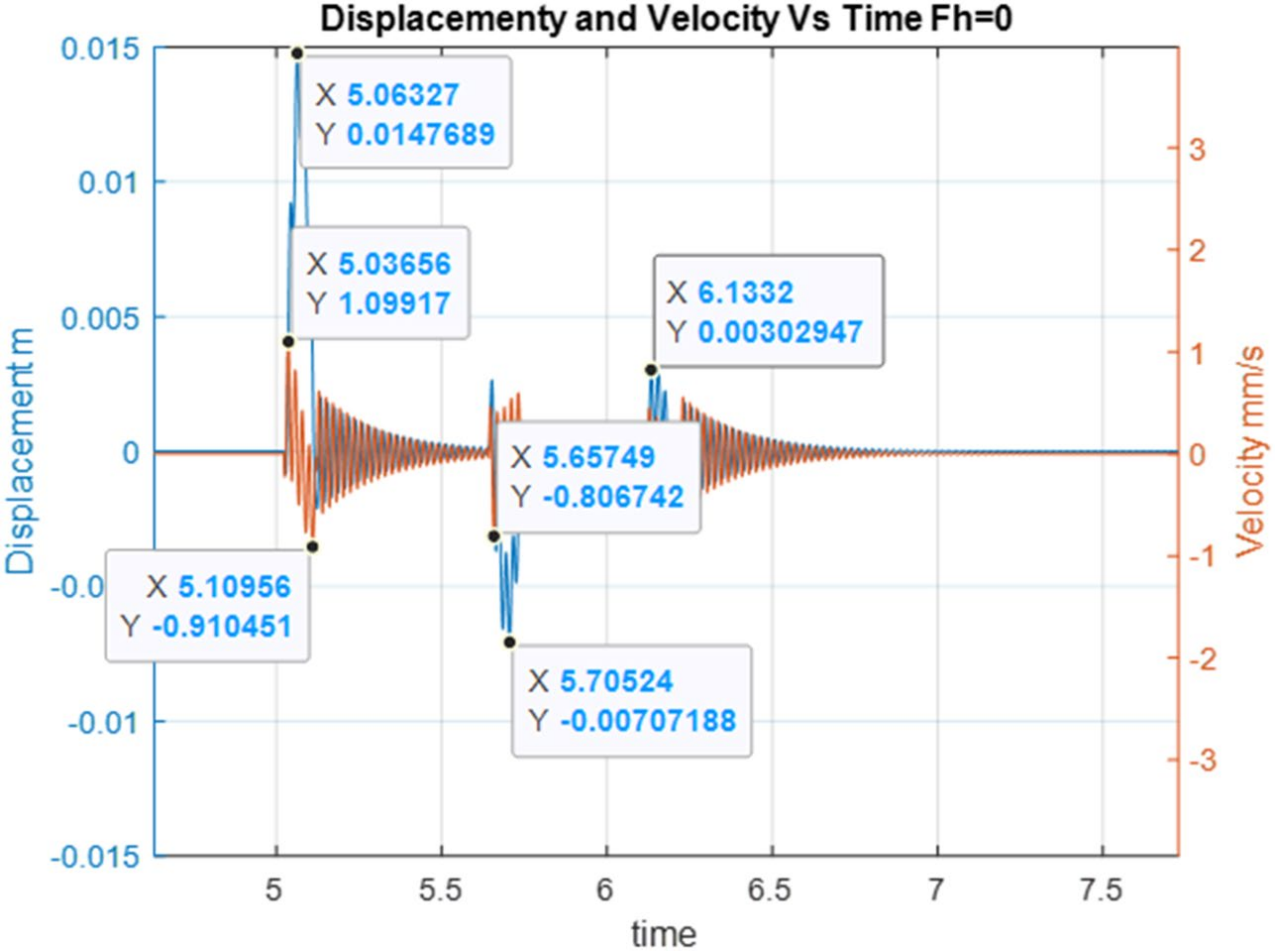
Displacement and velocity for extracted harmonic forces.

Below are the simulation results for various initial conditions—Figs. 4, 5, 6, 7, 8 and 9. The results were analysed to determine the size of the differences in the maximum displacement and velocity amplitudes at the first kick-off from the UNDEX impact. The results of the analysis are presented in Tables 1 and 2. For the simulation process, *Y*—means displacement, and *Y’*—means velocity for a specific impact shock initialisation time, which is the initial condition for the ODE simulation process. The min and max descriptions specify the minimum and maximum simulation initial conditions, respectively.

**Table 1:** Comparison result of displacement [m] during the simulation UNDEX impact.

Y’ = max	Y’ = min	Y = max	Y = min	Y = 0	F_harm_ = 0	Δmax	Difference %
0.022	0.02	0.021	0.019	0.014	0.014	0.008	36.36

**Table 2:** Comparison result of velocity (mm/s) during the simulation UNDEX impact.

Y’ = max	Y’ = min	Y = max	Y = min	Y = 0	F_harm_ = 0	Δmax	Difference %
3,89	3.47	4.23	5.069	1.59	1.09	3.979	78.49

The analysed results confirm that numerical simulations without external loads show significantly lower velocity and displacement vibration values for the analysed fan. All lowest values represent simulations without harmonic loads. The difference in the vibration speed reached as much as 78.49%.

Figure 4 shows an example of the model simulation result for ODE initial conditions with maximum velocity values (variant 1), and Fig. 5 shows minimum initial conditions (variant 2). The top speed for variant 1 was 12% higher, and the maximum displacement was 29% higher. Figures 6 and 7 (variants 3 and 4) show the simulation results for the ODE initial maximum and minimum displacement conditions, respectively (Y' = max, Y = 0 and Y' = min, Y = 0). In this case, the differences in displacement were less than 1%, while the difference in speed was higher by as much as 69% for variant 3. Figures 8 and 9 present comparative simulation results for the conditions Y' = 0 and Y = 0 (variant 5) and Y' = 0 and Y = 0 without harmonic forcing from the operating machine (variant 6). The results indicate much lower maximum displacement and speed values (the speed was only 37% compared to variant 3, and the displacement was 35%).

The statistical analysis results for different initial conditions with harmonic loads reach maximum differences of 27.27% for displacement and 31.36% for vibration velocity. The differences in displacement and vibration speed are 27.27% and 31.36%, respectively, for the selected example. During the simulation tests, several models were made for which the maximum differences reached 36.36% and 78.49%, respectively. This indicates the possibility of underestimating dynamic loads with simplified SDOF dynamic models.

## Laboratory tests

**Figure 10:**
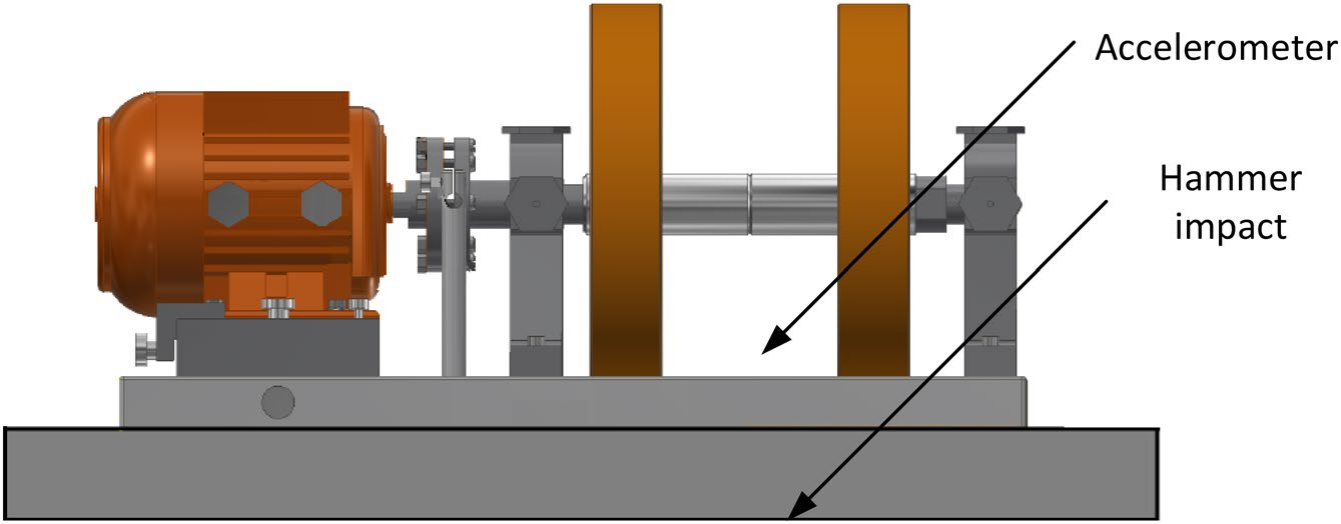
General view of the test object with the marked measuring point and the place of applying the force.

Verification of theoretical considerations was undertaken using a rotor machine. An electric motor drives the machine shown in Fig. 10, and its shaft has two concentrated masses in the form of rotating balanced discs. For the measurements, imbalances with a mass of 40 g were intentionally introduced to one of the disks, thanks to which a harmonic excitation with a frequency consistent with the rotational frequency of the rotor assembly shaft was obtained. An additional disturbance was introduced since the rotor used had only residual imbalance. Extra mass on the rotor disc simulated rotor unbalance and forced the harmonic excitation, which made the experiment closer to real conditions. For the measurements, unbalances with a mass of 40 g were intentionally introduced to one of the disks, thanks to which a harmonic excitation with a frequency consistent with the rotational frequency of the rotor assembly shaft was obtained. An additional disturbance was introduced since the rotor used had only residual imbalance. Additional mass on the rotor disc simulated rotor unbalance and forced the harmonic excitation, which made the experiment closer to real conditions. A marker was placed on one of the rotors to read the rotational speed, it was put in the place where the un-balancing mass was installed.

**Figure 11:**
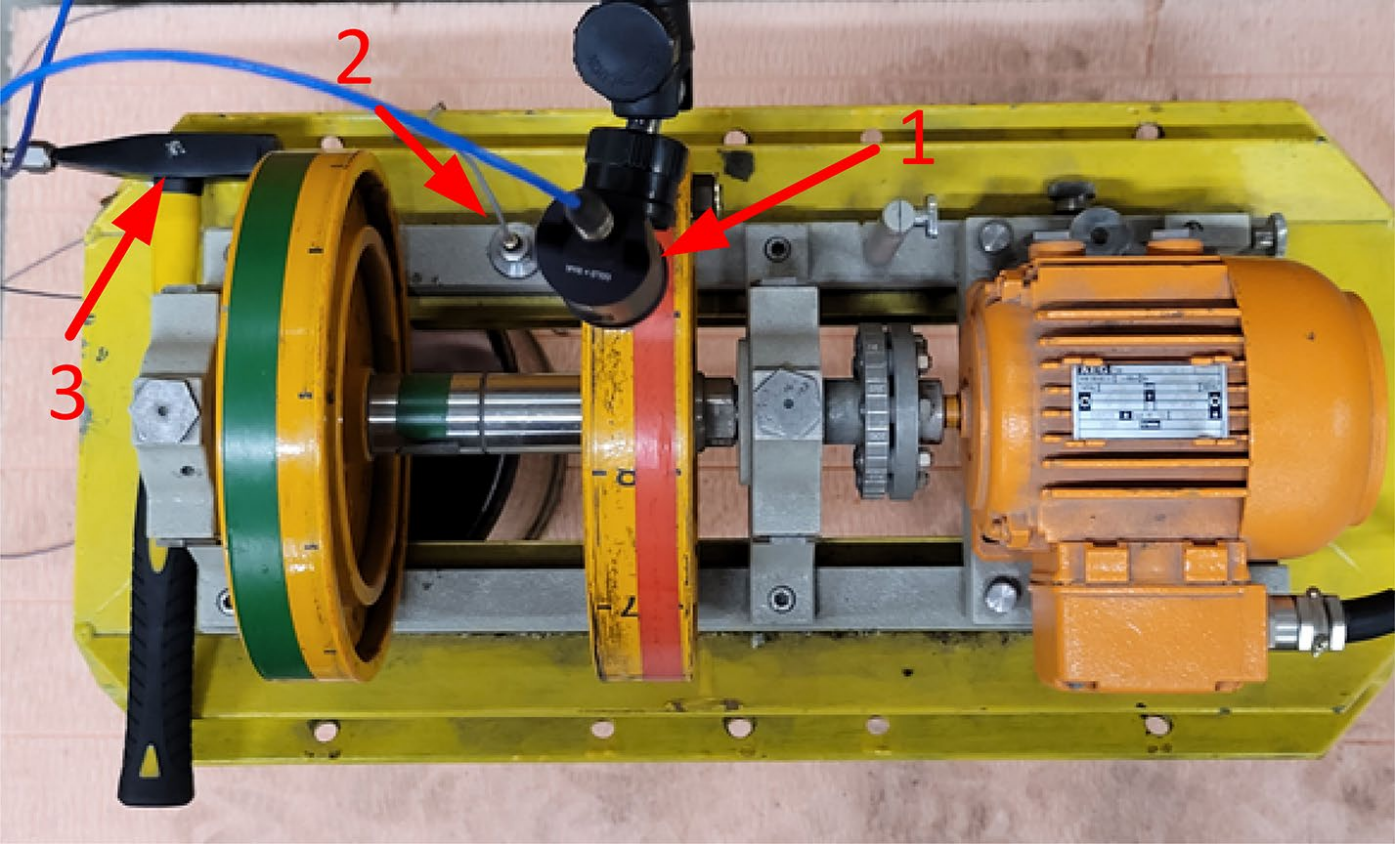
Measurement stand during tests. 1—tacho probe located in the upper position, 2—accelerometer, 3—hammer equipped with an accelerometer.

The research was carried out in two stages. In the first one, the laser rotational speed sensor was placed perpendicularly to the plane of the foundation of the rotor system from above and in the second from the bottom. The excitations with a hammer of 300 g mass, equipped with an accelerometer, were always applied in the same place, with the impact directed upwards of the tested system—Fig. 11.

This configuration made it possible to measure a situation where the inertia force associated with the non-equilibrium rotating mass was directed upwards and downwards. All signals were synchronised using one Bruel & Kjaer 3050-A-060 measurement system, two 4514B accelerometers and a 2981 type tachometric probe were used. The band from 0.7 to 3200 Hz with 8192 Hz sampling was recorded during the measurements.

**Figure 12:**
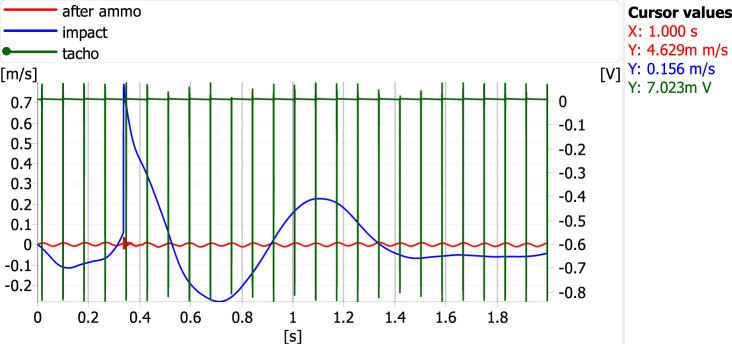
The entire length of time course record of the vibration velocity (red line—machine foundation, blue line—impact hammer) obtained from accelerometers with marked rotational speed markers (green line).

Several tests were made for both variants of the measurement. Among the registered signals, those were selected in which the excitation pulse from the hammer was as close as possible to the signal generated by the tag passing through the measurement area of the tachometer—Fig. 12. The harmonic nature of the excitation related to the unbalanced rotating mass is clearly visible on the charts (the course is marked in red).

**Figure 13:**
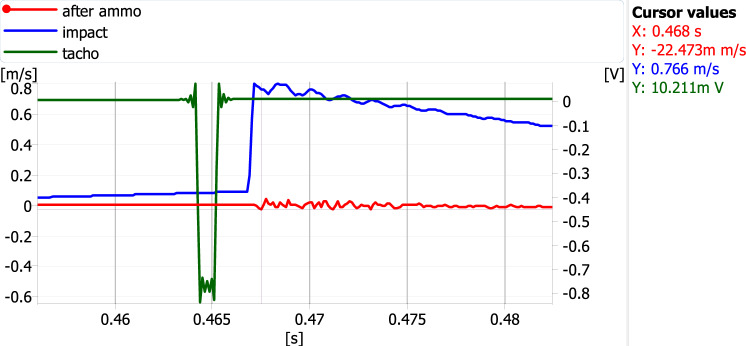
An example of signal time courses for the measurement variant when the vectors of the excitation force and the inertia force are in opposite directions.

**Figure 14:**
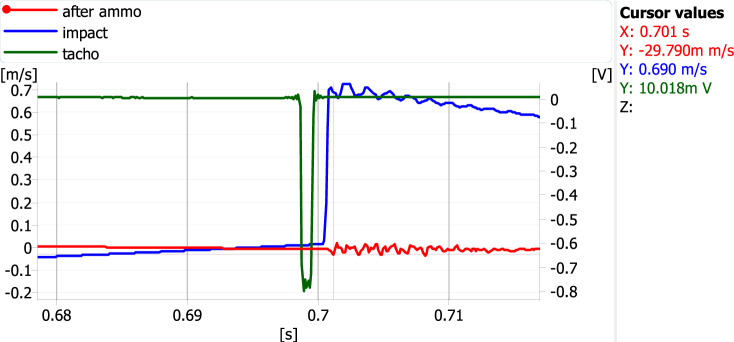
An example of signal time courses for the measurement variant when the excitation force and the inertia force vectors are in the same direction.

During the measurements, the acceleration of vibrations was recorded using piezoelectric accelerometers. The time courses of the accelerations were subjected to a single integration, as a result of which only the courses of the vibration velocity were used in the subsequent analyses. The values from the accelerometer mounted on the foundation of the machine (after ammo—red curves) and on the hammer with which the shock wave's impact was simulated (impact—blue curves) have opposite signs. This is because they were mounted in opposite directions—Figs. 13 and 14. For both measurement variants, five were selected in which the signal corresponding to the excitation simulating the shock wave was closest to the rotational speed marker. Examples of such waveforms are shown in Figs. 13 and 14.

All read values for both measurement variants are presented in Tables 3 and 4. The maximum value of the excitation velocity was read, followed by the absolute value of the first response maximum.

**Table 3:** Maximum excitation and response values when the vectors of the excitation force and the inertia force are in opposite directions.

Impulse amplitude (um/s)	Abs of response amplitude (um/s)	Speed ratio
776	21,689	35,778
821	19,738	41,595
615	14,980	41,055
736	18,510	39,762
806	22,473	35,865

**Table 4:** Maximum excitation and response values when the vectors of the excitation force and the inertia force are in the same direction.

Impulse amplitude (um/s)	Abs of response amplitude (um/s)	Speed ratio
797	36,532	21,816
811	34,712	23,364
698	29,243	23,869
706	22,783	30,988
735	27,343	26,881

The analysis of the data presented in Tables 3 and 4 and the waveforms of parameters in diagrams 13 and 14 clearly shows that if the direction of the impact of the unbalanced force vector resulting from the operation of the device is consistent with the direction of the impact of the force vector from the simulated underwater explosion, this results in an increase in the resultant velocity. A dimensionless coefficient called "speed ratio" was used to determine this impact in Tables 3 and 4. It is the ratio of the speed associated with the excitation from a simulated underwater explosion to the speed of the response recorded on the tested device (Fig. 11).

## Conclusions

The tests indicate that the fan's relative displacement depends on the initial conditions and external forces. The shock response analysis by the underwater explosion was performed on the 1D model with the charge mass and stand-off distance according to natural conditions. Significant differences in 1DOF results should prompt careful analysis of 3 DOF, especially under variable loads. Theoretical considerations and simulations were verified during laboratory tests. The performed numerical simulations indicate a significant influence of the initial conditions for the obtained shock impulse values for the naval rotation machines. The compounding of harmonic vibrations and shock impulse suggests the need to assume the most unfavourable load conditions of the device during the simulated UNDEX effect. The research shows that the safety coefficients used in the design of devices should be no less than K_dis_ = 1.4 for the displacement and K_vel_ = 1.8 for the vibration velocity.

Results obtained during the tests confirm the validity of the adopted theoretical assumptions and simulations. This work problem can be solved simplified by adjusting the safety factors or analytically by conducting more accurate studies of the occurring loads. Both available publications and normative documents dealing with the resistance of vessels to underwater explosions do not cover issues related to the tested machines' initial conditions. The simulations and laboratory tests carried out in this work confirm the existence of a significant influence of the direction of impact of forces resulting from the operation of the machine itself on the resultant force associated with an underwater explosion. Failure to consider this fact may lead to inappropriate conclusions, especially when safety factors with low values are adopted. Considering the existing threats, it seems more rational to use a detailed preliminary analysis for various operating conditions of devices and then carry out an SRS analysis.

## Data Availability

The datasets used and analysed during the current study are available from the corresponding author on reasonable request.
